# An Overview of the Randomized Placebo-Controlled Trials of Chinese Herbal Medicine Formula Granules

**DOI:** 10.1155/2019/6486293

**Published:** 2019-04-11

**Authors:** Mei Han, Lily Lai, Xin-xue Li, Nan-qi Zhao, Jing Li, Yun Xia, Jian-Ping Liu

**Affiliations:** ^1^School of Traditional medicine, Beijing University of Chinese Medicine, China; ^2^Primary Care and Population Sciences, University of Southampton, UK; ^3^World Federation of Chinese Medicine Societies, China; ^4^Dongfang Hospital Beijing University of Chinese Medicine, China

## Abstract

**Objective:**

To summarize the characteristics and the outcomes of the Randomized Placebo-Controlled Trials of Chinese Herbal Medicine Granules manufactured by China Resources Sanjiu Pharmaceutical Co., Ltd.

**Methods:**

Databases including China National Knowledge Infrastructure, VIP, Wanfang, PubMed, Cochrane Library, and clinicaltrials.gov were searched in March 2018 for relevant randomized controlled trials (RCTs). Two reviewers independently screened for and selected studies, extracted data, and checked data extraction. Methodological quality was evaluated using the Cochrane Risk of Bias tool. For the outcome, the characteristics of the study, the cure rate, the effectiveness rate, and advert events were described with a method of bibliometrics. Also, we performed meta-analysis only if there were ≥2 studies treated by the same intervention and evaluated by the same outcome.

**Results:**

A total of 40 placebo-controlled RCTs treated for 17 diseases were included in our analysis involving 4,632 patients. 16 of 19 studies treated by CHM granules only showed positive result in patients with HBV, HCV, fever, depression, nonalcoholic fatty liver disease, AIDS, and asthma while negative result was shown in patients with migraine. 17 of 21 studies treated by combination therapy against conventional therapy showed positive result in patients with HBV, herpes simplex keratitis, COPD, liver cirrhotic ascites, Parkinson's disease, and diabetic peripheral neuropathy while negative result was shown in patients with myasthenia gravis, angina pectoris, and depression. The pooled result cannot demonstrate that the notifying kidney formula granules had the superior effect with placebo on the clearance of serum HBV DNA and HBeAg in HBV carriers with a RR (and the 95% CI) of 2.97 [0.74,11.91] and 1.99 [0.93,4.29], respectively. But, the CHM granules can reduce within-group HBV DNA levels by more than 2 lgIU/ml; the RR (and 95% CI) was 4.64 [2.89,7.45]. Qizhu granules had a significant effect on clearance of HCV RNA with a RR (and 95% CI) of 6.26 [2.16,18.16]. And, the heat-clearing and detoxifying formula granules were superior to placebo in resolution of cold symptom among patients with fever with a RR and 95% CI of 2.58 [1.40,4.74]. Based on the conventional therapy, the pooled result demonstrated that the Regulating liver formula granules were superior to placebo on the clearance of serum HBeAg in chronic hepatitis B patients with a RR (and the 95% CI) of 1.73 [1.30,2.31]. The EeChen decoction granules were superior to placebo in COPD patients with a RR (and the 95% CI) of 1.13 [1.06,1.22]. 28 of the 40 studies reported adverse events. There were 51 adverse events in CHM formula granules group or combination group (n=2,483) and 26 in control group (n=2,122) totally. Most of the adverse symptoms spontaneously resolved after completing the courses of treatment and the other adverse symptoms improved after symptomatic treatment.

**Conclusion:**

16 of 19 studies treated by CHM granules only showed positive result in 7 diseases and negative result in 1 disease. 17 of 21 studies treated by combination therapy against conventional therapy showed positive result in 6 diseases and negative result in 3 diseases. However, both the absolute and relative effectiveness of CHM formula granules compared with placebo need to be considered clinically.

## 1. Introduction

Chinese herbal medicine (CHM) formula granules are concentrated extracts of Chinese herbs which is a convenient and increasingly popular method of administering Chinese herbal medicine (CHM) [1]. Traditionally, CHMs are prescribed as dried herbs that are decocted daily with water by the patient and could be time-consuming. Manufacturers claim that CHM formula granules are superior to traditional decoction methods in terms of the ease of administration which improves patient adherence to treatment and retains the properties of CHMs to maintain effectiveness [2].

In the early 2000s, traditional Chinese medicine became increasingly industrialized and has continued to develop rapidly and widely with increasing investment into developing CHM formula granules [3]. Given that traditional use of CHMs has typically involved decoctions, research on CHM formula granules has increasingly focused on its equivalence with decoction methods, whether it is the chemical composition of CHM formula granules or clinical efficacy [2]. Comparative studies of chemical composition have compared single Chinese herbs as well as CHM formula granules and although the differences are clear in terms of chemical constituents, the conclusions drawn from these studies are varied [4]. Looking at the available clinical research, however, most studies appear to show that formula granules are equivalent to that of traditional decoction methods [4–6], but a few studies show the traditional decoction methods were better in some outcomes for some diseases [4, 7].

Compared with traditional decoction methods, research into the efficacy and safety of CHM formula granules is limited, despite growing popularity of its use. We aimed to describe the status and summarize the outcomes of the studies treated by CHM formula granules compared to placebo by conducting a research of bibliometrics and meta-analysis.

## 2. Methods

### 2.1. Inclusion/Exclusion Criteria

We only included RCTs and accepted interventions involved CHM formula granules compared with placebo granules. We placed no limits on the topic of disease of the research. The intervention group is treated by CHM formula granules only or combined with conventional therapy, and the control group is treated by CHM formula placebo granules or combined with conventional therapy. We placed no limits of the outcomes. Since there is currently no national quality standard for CHM formula granules, the quality of CHM formula granules can differ among manufacturers [8]. For this reason, only studies involving the most widely used CHM formula granules were accepted. These are manufactured by China Resources Sanjiu Pharmaceutical Co., Ltd., which is one of the six manufacturers first certified by the National Food and Drug Administration and supplies the largest number of terminal hospitals in China [3].

### 2.2. Literature Search

We searched for published literature in databases including China National Knowledge Infrastructure (CNKI), Wanfang Data, Chong Qing VIP, PubMed, and Cochrane Library. We retrieved completed but unpublished studies from clinicaltrials.gov and tracked the results of these studies. Only Chinese and English articles were retrieved, and the last search was carried out on 31st March 2018. The search words included “CHM formula granules”, “Chinese Herbal Medicine granules”, “dispensing granules”, “traditional Chinese medicine particle”, “pellet formula of traditional Chinese medicine”, “concentrated granule”, “TCM formula granule”, “TCM formula particles”, “Sanjiu”, “999”, and “placebo”. Depending on the characteristics of each database, search strategies including keywords or full phrases were used.

### 2.3. Literature Screening

Identified articles were initially imported into NoteExpress and initial screening carried out based on inclusion/exclusion criteria after reading article titles and abstracts. For the next stage of screening, full texts were acquired and checked for eligibility before including articles in the final analysis.

### 2.4. Quality Evaluation

The risk of bias was assessed according to the Cochrane Handbook for Systematic Reviews of Interventions [9], which involves assessing bias relating to random sequence generation, allocation concealment, blinding, data integrity, selective reporting of positive and/or negative findings, and other sources of bias. Among them the “other sources of bias” (a) were the clear inclusion/exclusion criteria; (b) were the baseline data comparable; and (c) was any conflict of interest. For selective outcome reporting, since most of the included studies did not register their research protocols, the risk of bias was defined as low if the outcome measures included all the important outcomes according to the target diseases of the original studies. The risk of bias was assessed and validated independently by two reviewers (Mei Han and Jing Li); the results were cross-referenced, and any disagreements were resolved by discussion with a third reviewer (Jian-ping Liu).

### 2.5. Outcome Measures

The cure rate (percentage of the total number of people cured) and the overall effectiveness rate (percentage of the total number of people improved) were described. Other indicators of effectiveness will be described if there is no information of effective rate and cure rate; and adverse events as the key safety indicator will be described as well. The cure rate and the overall effectiveness rate are calculated with the data from the included studies according to their standard method for clinical evaluation of therapeutic effects.

### 2.6. Data Analysis

Descriptive statistics (frequency and percentage) were used to describe the characteristics and the outcome of the studies. Statistical pooling was carried out using the Cochrane Collaboration software RevMan5.3.5 for meta-analysis only if there were ≥2 studies treated by the same intervention and evaluated by the same outcome. The relative risk (RR) and 95% confidence interval (CI) were selected as the statistic for dichotomous data. To evaluate heterogeneity, chi-square test was first performed and based on this finding; estimates of heterogeneity (I^2^) were then applied. We used a fixed-effects model when I^2^ was ≤50% and the P value was ≥0.10; when I^2^ was >50% or the P value was <0.10, we applied a random-effects model.

## 3. Results

### 3.1. Literature Searching and Screening Flowchart

**Figure 1:**
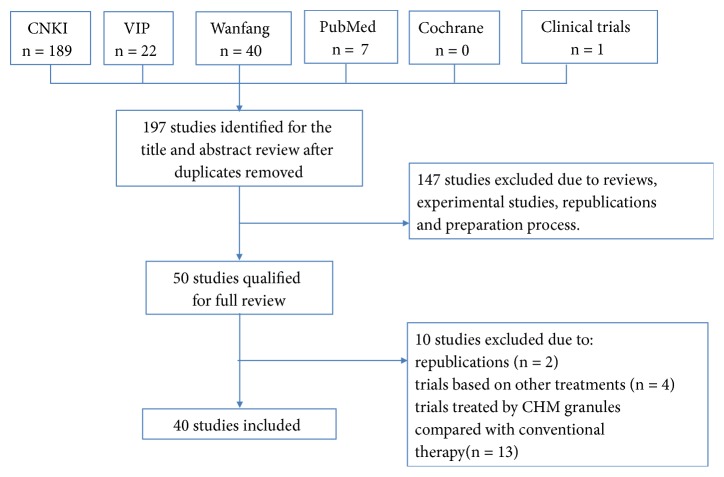
Flowchart of study searches and screening.

252 articles were retrieved in total based on our search strategies. After removing duplicates and screening titles and abstracts, we obtained 50 full-text articles. 40 articles were eventually eligible for inclusion in this review (Figure 1).

### 3.2. Characteristics of Included Literature

A total of 40 RCTs involving 4632 patients [10–49] were included. 19RCTs compared the effects of CHM formula granules alone versus placebo granules and involved the treatment of 8 conditions. These conditions include chronic hepatitis B or hepatitis B virus (HBV) carrier [11, 18, 19, 21, 22, 24, 28], chronic hepatitis C [27, 49], nonalcoholic fatty liver disease [25], migraine [14–16], fever [10, 11], depression [25, 26], acquired immune deficiency syndrome (AIDS) [12], and asthma [17]. 21 RCTs compared the effects of CHM formula granules versus placebo granules based on conventional therapy and involved the treatment of 9 conditions. These conditions include Parkinson's disease [32–40], COPD [28–30], chronic hepatitis B [45–47], diabetic peripheral neuropathy [31], depression [41], angina pectoris [42], myasthenia gravis [43], herpes simplex keratitis [44], and liver cirrhotic ascites [48].

The total composition of the CHM formula was described in 37 studies [10–45, 48, 49] and, among them, 32 studies described the dosage for each kind of the granule. 28 studies described the ingredients of the placebo which included a small dose of CHM formula granules in 6 studies [10, 11, 13, 27, 28, 49], the Chinese herb Huo Xiang (wrinkled giant hyssop) in 6 studies [18, 20, 21, 23, 24, 45], and no pharmaceutical ingredients in 3 studies [17, 25, 26, 31–44, 48]; also, the placebo contains some food colorings, starch, maltodextrin, or anhydrous glucose. The specific characteristics of these studies were summarized in Table 1.

### 3.3. Quality Evaluation of the Articles

**Figure 2:**
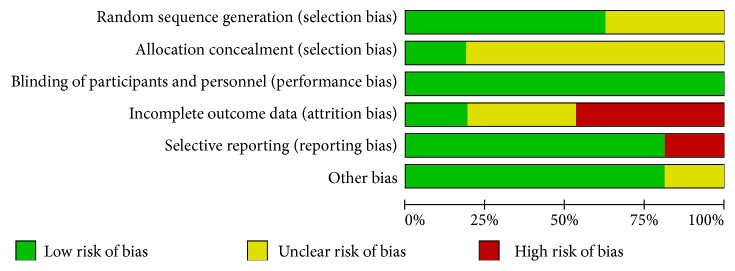
Evaluation of the risk biases of the included studies.

Among these 40 studies, patients were randomized by random number table in 13 studies [10, 11, 13, 17, 25, 26, 31, 41–45, 48], by a central randomization system in 13 studies [15, 18, 19, 28, 32–41] and by using SAS software in 1 study [21]; the remaining 13 studies only mentioned “random” or “randomization” without describing the specific randomization methods [12, 14, 16, 20, 22, 24, 27–30, 46, 47, 49]. 14 studies described allocation concealment with the central randomization system [15, 18, 19, 21, 23, 32–41]. One study described the blinding only of participants [17] and the remaining 38 studies blinding of both participants and researchers. 28 studies described the number of participants who withdrew and were lost to follow-up and only 6 of these provided reasons [10, 13, 21, 23, 26, 31]. The number of patients in all the randomized groups and in the analysis was the same in the remaining 11 studies. The research protocol was registered before publication in only 1 study [12] and all the important outcomes were reported in 26 studies without selective outcome reporting [10, 11, 13–19, 25–31, 39–43, 49]. The remaining 14 articles only reported part of the important outcomes [11, 20–22, 24, 28, 32–41]. 32 studies were supported by the Chinese government project [12–14, 16–23, 25–28, 28, 29, 29–38, 42, 44–49] and the remaining 8 were unknown. The quality of the studies included in our analysis was not bad. The details of the evaluation were shown in Figure 2.

### 3.4. Results

#### 3.4.1. Cure Rate

14 of the 19 studies treated by CHM formula granules only, and 6 of the 21 studies treated by CHM formula granules combined with conventional therapy described the cure rate. While the definition of “cure” differed among these studies with different conditions, the standard for clinical evaluation of therapeutic effects was described in detail as follows.

According to the outcome “cure rate”, 50.00% of the studies treated by CHM formula granules only showed a positive result, such as chronic hepatitis C, nonalcoholic fatty liver disease, depression, and fever, and another 50% studies showed a negative result, such as HBV carrier, HBV recrudescence, and migraine. Four studies treated by the notifying kidney formula evaluated the effects on HBV carriers. The pooled result of 4 studies cannot demonstrate that the notifying kidney formula granules had the superior effect with placebo on the clearance of serum HBV DNA and HBeAg in HBV carriers with a RR (and the 95% CI) of 2,97 [0.74,11.91] and 1.99 [0.93,4.29], respectively. Pooled analysis of 3 studies showed that Qizhu granules had a significant effect on clearance of HCV RNA with a RR (and 95% CI) of 6.26 [2.16,18.16]. The pooled result showed the heat-clearing and detoxifying formula granules were superior to placebo in resolution of cold symptom among patients with fever with a RR and 95% CI of 2.58 [1.40,4.74].

Among the 6 combination studies reporting cure rate, only 1 study treated for herpes simplex keratitis showed positive results in 2 subgroups. The pooled result demonstrated that the Regulating liver formula granules were superior to placebo on the clearance of serum HBeAg in chronic hepatitis B patients based on the conventional therapy with a RR (and the 95% CI) of 1.73 [1.30,2.31], but the formula did not work with clearance of serum HBV DNA. The negative results also were shown in the studies treated for myasthenia gravis and depression. The results of all the individual outcome and meta-analyses carried out are shown in Tables 2 and 3.

#### 3.4.2. Clinical Effectiveness Rate

12 of these 19 studies treated by CHM formula granules only, and 8 of the 21 studies treated by CHM formula granules combined with conventional therapy compared the clinical effectiveness rate. As with the “cure” rate, the definition of “clinical effectiveness” differed among studies with different conditions. Again, the standard for clinical evaluation of therapeutic effects of the different conditions is described in detail below.

According to the outcome “clinical effectiveness rate”, 75.00% of the studies treated by CHM formula granules only showed a positive result, such as HVB carrier, nonalcoholic fatty liver disease, depression, and AIDS, and another 25% studies showed a negative result, such as fever and migraine. Five studies treated by the notifying kidney formula evaluated the effects on HBV carriers. Pooled analysis showed that the granules can reduce within-group HBV DNA levels by more than 2 lgIU/ml; the RR (and 95% CI) was 4.64 [2.89,7.45]. There was no significant difference between heat-clearing and detoxifying formula granules and placebo granules to improve fever and cold symptoms with a RR (and 95% CI) of 3.78 [0.50,28.36].

Among the 8 combination studies reporting effectiveness rate, 1 study treated for diabetic peripheral neuropathy showed positive results. The pooled result of 3 studies demonstrated that the EeChen decoction granules were superior to placebo in COPD patients based on the conventional therapy with a RR (and the 95% CI) of 1.13 [1.06,1.22]. The negative results were shown in the studies treated for myasthenia gravis, angina pectoris, herpes simplex keratitis, and depression. The results of all the individual outcome and meta-analyses carried out are shown in Tables 4 and 5.

#### 3.4.3. Overview of Effectiveness of CHM Formula Granules

According to the studies included, we calculated the overall cure rate and effectiveness rate of the diseases. It showed that, in some disease, there was poor effectiveness even if there was significant difference between groups. The results are shown in Tables 6 and 7.

Neither cure rate or effectiveness rate was not described in 10 studies; among these studies, 1 study treated by CHM granules only compared with placebo in patients with asthma showed fewer attacks in the CHM granules group (P<0.05) [17]. One study treated by CHM granules combined with conventional therapy compared with conventional therapy only in patients with liver cirrhotic ascites showed undifferentiated mortality (4/56 versus 9/56, p=0.16) and lower 1-year recurrence rate (25/56 versus 41/56, p=0.003) [48]. The other 8 studies were conducted by the same research team and each study reported the outcomes selectively. The research showed that notifying the kidney and promoting blood circulation formula combined with madopar can improve the sleep quality, motor function, muscle tension, dopamine level, and TCM syndromes in patients with Parkinson's disease [32–40].

#### 3.4.4. Adverse Events

28 of the 40 studies reported adverse events. Among them, 8 studies [10, 11, 13, 15, 17, 21, 27, 28] reported that there was no adverse event. In the migraine study [14], nausea, abdominal pain, diarrhea, abdominal distention, and constipation appeared in 15 participants, 10 in the CHM formula granules group and 5 in the placebo group. In the 7 chronic hepatitis B studies [18, 20, 22–24, 45, 47], slight abdominal distention, loss of appetite, and abdominal discomfort appeared in 28 participants, 21 in the CHM formula granules group, and 7 in the placebo group. In addition, mildly raised ALT levels were found in 8 participants in the CHM formula granules group. In the 3 depression studies, dry mouth, nausea, abdominal distention, and dizzy were found in 25 participants, 15 in the CHM formula granules group, and 10 in the placebo or conventional therapy group. In the herpes simplex keratitis study, nausea and diarrhea were found in 4 patients in the CHM granules group. In the 8 with Parkinson's disease, pneumonia was found in 1 participant in the CHM granules group, and stomachache in 2 participants, fever in 1 participants, and slightly cerebral infarction in 1 participant in the control group [28–35]. There were 51 adverse events in CHM formula granules group or combination group (n=2,483) and 26 in control group (n=2,122) totally. All adverse symptoms spontaneously resolved after completing the courses of treatment and the participants with mildly raised ALT levels, slightly cerebral infarction, and pneumonia improved after symptomatic treatment.

## 4. Discussion

### 4.1. Principal Findings

Based on the findings in this systematic review, positive result was found in studies treated by CHM granules only for patients with HBV/HCV, nonalcoholic fatty liver disease, depression, fever, asthma, and AIDS and negative result for patients with migraine. It showed significant difference in studies for patients with HBV, HCV, depression, and fever by meta-analysis. Despite this, levels of HBV DNA and HBeAg clearance for CHM formula granules were much lower than for antivirals [50] or interferon [51]. This is a similar finding for patients with HCV RNA and HCV RNA clearance levels for CHM formula granules were much lower than for antivirals [52].

Also, positive result was found in studies treated by CHM granules combined with conventional therapy for patients with HBV, herpes simplex keratitis, COPD, liver cirrhotic ascites, Parkinson's disease, and diabetic peripheral neuropathy and negative result for patients with myasthenia gravis, angina pectoris, and depression. It showed significant difference in studies for patients with HBV and COPD by meta-analysis.

Owing to the low incidence of adverse events and high spontaneous resolution of adverse events, CHM formula granules produced by China Resources Sanjiu Pharmaceutical Co., Ltd., can be regarded as being clinically safe.

### 4.2. The Limitations of These Studies

The limitation of our research is that we only included studies of CHM formula granules produced by China Resources Sanjiu Pharmaceutical Co., Ltd. However, it was our view that the granules produced by different manufacturers would be of varying quality and effectiveness which would make it difficult for us to be able to reduce clinical heterogeneity. For this reason, we chose granules produced by Sanjiu which are the most widely among hospitals in China. Finally, the 40 studies included in this review evaluated 17 conditions and the pooled result of each subgroup may not be particularly reliable. Despite this limitation, our view was that producing this systematic review would enable us to map the current evidence for placebo-controlled RCTs investigating CHM formula granules.

### 4.3. Implications for Clinical Practice and Further Research

The results of our systematic review suggest that CHM maybe is a safe treatment. CHM formula granules alone are potentially able to benefit patients with depression and may lead to a quicker resolution of symptoms for patients with fever. However, the current evidence suggests that CHM formula granules alone are not suitable for viral clearance of HBV or HCV. CHM formula granules combined with conventional therapy can improve the symptoms of patients with COPD and help viral clearance of HBV in patients with chronic hepatitis B.

The pooled results were limited by the small number of studies for the same condition and relatively small sample sizes. We recommend that further research to evaluate the clinical effectiveness of CHM formula granules be carried out by carrying out a larger number of placebo-controlled RCTs for the same condition and which should be adequately powered. Future studies should also focus on the choice of the endpoint to ensure that clinically relevant and internationally validated outcomes are incorporated.

## 5. Conclusion

In this systematic review of 40 randomized placebo-controlled trials, 19 studies treated by CHM granules only showed positive result in patients with HBV, HCV, fever, depression, nonalcoholic fatty liver disease, AIDS, and asthma and negative result in migraine. 21 studies treated by combination therapy showed positive result in patients with HBV, herpes simplex keratitis, COPD, liver cirrhotic ascites, Parkinson's disease and diabetic peripheral neuropathy and negative result in patients with myasthenia gravis, angina pectoris, and depression. And, the results of our systematic review suggest that CHM granules maybe is a safe treatment. However, it is important to consider the clinical relevance of both the absolute and relative effectiveness of CHM formula granules compared with placebo granules in order to maximize the relevance of these findings to patients, medical professionals, and commissioners.

## Figures and Tables

**Table 1:** Characteristics of the included studies.

Study ID	Diseases	Sample T/C	Age (years)	Gender M/F	Treatment	Duration	Main outcomes
Cheng Y 2016 [10]	fever	41/40	T:38.03 ± 14.65 C:37.21 ± 15.43	T:22/19 C:20/20	Clearing heat and removing toxicity formula	3 d	Fever clearance time, AE

Xie JH 2017 [11]	fever	48/48	T:36.7 ± 6.7 C:39.4 ± 2.1	T:25/23 C:20/28	Clearing heat and removing toxicity formula	3 d	symptoms improving, AE

Li Y 2012 [12]	AIDS	36/36	18-70	46/26	Improving immunity formula	180 d	CD4

Cheng Y 2017 [13]	non-alcoholic fatty liver	48/48	T:45.07 ± 11.23 C:41.65 ± 9.38	T:23/17 C:25/15	Invigorating spleen, regulating Qi and resolving dampness formula	24 w	Free fatty acids, ALT, AE

Fu CH 2014 [14]	migraine	150/78	T:37.11 ± 11.96 C:34.97 ± 10.40	T:33/96 C:12/52	Modified *Rhizoma Chuanxiong* releasing pain decoction or *Wuzhuyu* decoction	12 w	No. and degree of headache

Xu YL 2011 [15]	migraine	24/24	18~65	T:5/19 C:11/13	Regulating liver, dispelling wind and removing blood stasis formula	12 w	No. and degree of headache, AE

Zhang Y 2013 [16]	migraine	86/42	T:23 ~ 56 C:24 ~ 54	T:23/63 C:11/31	Modified Chuanxiong Dingtong decoction	12 w	No. of headache, PSQI scale, EO

Geng YY 2015 [17]	asthma	40/20	T:3.62 ± 0.88 C:3.37 ± 0.90	T:30/10 C:17/3	Nourishing Qi to invigorate spleen and invigorating the kidney formula	12 w	No. of asthma attacks, AE

He JS 2012 [18]	HBV carrier	200/100	T:33.6 ± 7.1 C:34.4 ± 8.2	T:137/63 C:60/40	Notifying kidney and spleen formula	52 w	HBV DNA, HBeAg, HBsAg, AE

Li HZ 2012 [19]	HBV carrier	200/100	T:34.33 ± 6.22 C:34.39 ± 8.18	T:118/82 C:59/41	Notifying and clearing kidney formula	52 w	HBV DNA, HBeAg

Ni W 2015 [20]	HBV carrier	40/20	36. 24 ± 8. 22	T:19/21 C:12/8	Notifying and clearing kidney formula	48 w	HBV DNA, HBeAg, AE

Peng DT 2016 [21]	HBV carrier	50/25	T:34.67 ± 5.12 C:32.20 ± 3.95	T:24/26 C:14/11	Notifying kidney and detoxifying formula	48 w	HBV DNA, HBeAg, HBsAg, AE

Xing YF 2012 [22]	HBV carrier	200/200	T:34.33 ± 6.22 C:34.39 ± 8.18	T:113/78 C:112/75	Notifying and clearing kidney formula	52 w	HBV DNA, AE

Zheng YJ 2012 [23]	HBV carrier	60/60	T:35.0 ± 5.0 C:34.0 ± 6.0	T:42/18 C:45/15	Notifying kidney and spleen formula	48 w	HBV DNA, HBeAg, AE

Zhang ZE 2013 [24]	HBV recrudescence	23/23	T:37.7 ± 12.5 C:38.1 ± 12.0	T:12/11 C:13/10	Notifying kidney and spleen formula	48 w	HA, PCIII, AE

Xu F 2013-1 [25]	depressed	50/50	T:30.43 ± 3.43 C:30.07 ± 3.34	-	Benefiting brain and relieving depression formula	6 w	Edinburgh Postnatal Depression Scale, AE

Xu F 2013 [26]	depressed	47/46	-	-	Ease powder	6 w	cognitive function, AE

Zhao L 2014 [16]	HCV	102/102	1b:54.7 ± 12.47 other:49.5 ± 16.7	T:81/48 C:24/20	Qizhu (*astragalus* and *atractylodes*) granules	48 w	HCV RNA

Zhao L2014-1 [27]	HCV	96/96	T:53.47 ± 12.80 C:53.27 ± 13.24	T:39/57 C:34/52	Qizhu (*astragalus* and *atractylodes*) granules	48 w	HCV RNA, AE

Chen SQ 2017 [28]	COPD	60/60	T:65.06 ± 5.05 C:66.08 ± 6.04	T:29/31 C:32/28	Modified *Erchen decoction* combined with antibiotics, bronchiectasis and oxygen uptake	14 d	Pulmonary function, IgA, AE

Xie WY 2017 [29]	COPD stable period COPD acute period	60/60 60/60	T:65.05 ± 5.07 C:68.07 ± 6.15 T:68.12 ± 5.07C:68.11 ± 6.06	T:33/27 C:31/29 T:32/28 C:30/30	Modified *Erchen decoction* combined with antibiotics, bronchiectasis and oxygen uptake	14 d	Pulmonary function

Shang LZ 2017 [30]	COPD	100/100	T:69.48 ± 9. 05C:70.39 ± 8. 84	T: 57/43 C: 55/45	Modified *Erchen decoction* combined with antibiotics, bronchiectasis and oxygen uptake	14 d	Pulmonary function

Hu WH 2015 [31]	diabetic peripheral neuropathy	50/50	T:54.5(43-68) C:53.2(46-69)	T:24/26 C:22/28	Eliminating arthralgia and promoting blood circulation granules combined with methycobal and antidiabetics	8 w	Pain, neurological function

Li M & Yang MH [32–40]	Parkinson's disease	60/60	T:66.6 ± 1.2 C:67.3 ± 1.2	T:31/27 C:42/20	Notifying the kidney and promoting blood circulation formula combined with madopar	36 w	Unified Parkinson's disease rating scale, AE

Liu J 2012 [41]	depression	30/30	T:40.13 ± 9.72 C:41.25 ± 9.59	T:14/18 C:13/20	Relieving depression granules combined with paroxetine	8 w	HAMD Scale, AE

Pang LJ 2013 [42]	angina pectoris	35/35	T:57.2 ± 9.2 C:60.13 ± 3.8	T:15/15 C:15/15	Promoting blood circulation and resolving turbidity formula combined with aspirin	8 w	Pain, attacks, ECG, nitro-glycerine

Shuang XP 2014 [43]	myasthenia gravis	20/18	T:22-60 C:20-59	T:8/12 C:7/11	Notifying Qi and dehumidification formula combined with prednisone and pyridine bromide	10 w	Myasthenia gravis scale

Song JK 2016 [44]	herpes simplex keratitis (liver-wind and deficiency of liver-Yin)	35/40 38/40	T:36.5 ± 7.3 C:38.2 ± 8.6 T:46.5 ± 8.2 C:44.2 ± 7.6	T:17 /16 C:16 /15 T:15/17 C:16 /18	Clearing or softening liver formula combined with ganciclovir	7 d	Symptoms, laboratory test, AE

Xie WN 2016 [45]	HBV	40/40	T:27.43 ± 5.75 C:28.72 ± 6.42	T:30/10 C:31/9	Notifying kidney and spleen formula combined with lamivudine	48 w	HBV DNA, HBeAg, AE

Ye YA 2012 [46]	HBV	295/295	-	-	Regulating liver and blood and notifying spleen/Regulating liver, detoxification and dehumidification combined with adefovir dipivoxil	48 w	HBeAg

Zhan BL 2013 [47]	HBV	30/30	T:34.26 ± 9.41 C:36.19 ± 10.29	T:21/9 C:19/11	Regulating liver and blood and notifying spleen/Regulating liver, detoxification and dehumidification combined with adefovir dipivoxil	48 w	HBV DNA, HBeAg, AE

Zhang PJ 2013 [48]	liver cirrhotic ascites	56/56	T:42.7 ± 6.42 C:44.72 ± 5.48	T:32/24 C:30/26	Alleviating water retention formula	28 d	Liver function

*∗* AE: adverse event; -: undescribed.

**Table 2:** Comparison of the cure rate between CHM formula granules and placebo.

Study ID	Treatment		Placebo		P value
	n/N	%	n/N	%	
*HBV carrier: HBV DNA negative after 48-week treatment*					
He JS 2012	1/174	0.57	0/93	0.00	0.77
Li HZ 2012	3/191	1.57	0/94	0.00	0.35
Ni W 2015	3/40	7.50	0/20	0.00	0.41
Xing YF 2012	3/191	1.57	1/187	0.53	0.38
Meta-analysis RR and 95% CI: 2.97 [0.74,11.91]; I^2^ = 0%					

*HBV carrier: HBeAg negative after 48-week treatment*					
He JS 2012	11/174	6.32	3/93	3.23	0.29
Li HZ 2012	7/191	3.66	3/94	3.19	0.84
Ni W 2015	5/40	12.50	1/20	5.00	0.38
Zheng YJ 2012	6/56	10.71	2/57	3.51	0.15
Meta-analysis RR and 95% CI: 1.99 [0.93,4.29]; I^2^ = 0%					

*HBV recrudescence: HBV DNA negative after 48-week treatment*					
Zhang ZE 2013	9/23	39.13	1/23	4.35	0.02

*HBV recrudescence: HBeAg negative after 48-week treatment*					
Zhang ZE 2013	7/23	30.43	2/23	8.70	0.08

*Chronic hepatitis C: HCV RNA negative after 48-weektreatment and 24-week follow-up*					
Zhao L 2014 (HCV-1b)	4/44	9.09	0/51	0.00	0.11
Zhao L 2014 (HCV-other)	6/14	42.86	1/15	6.67	0.04
Zhao L 2014-1	10/60	16.67	3/68	4.41	0.03
Meta-analysis RR and 95% CI: 6.26 [2.16,18.16]; I^2^ = 0%					

*Non-alcoholic fatty liver disease: Normal ALT and B-ultrasound after 12-week treatment*					
Cheng Y 2017	19/40	47.50	10/40	25.00	0.04

*Depression: Reduction in Edinburgh Postnatal Depression Scale (EPDS)≥80*%					
Xu F 2013-1	13/50	26.00	4/50	8.00	0.02
Xu F 2013	14/45	31.11	6/45	13.33	0.05

*Fever: Normal temperature and resolution of cold symptoms*					
Cheng Y 2016	27/41	65.85	18/40	45.00	0.06
Xie JH 2017	29/48	60.42	17/48	35.42	0.02
Meta-analysis RR and 95% CI: 2.58 [1.40,4.74]; I^2^ = 0%					

*Migraine: Resolution of symptoms after 12-week treatment and at 2-month follow-up*					
Xu YL 2011	4/24	16.67	0/24	0.00	0.12

**Table 3:** Comparison of the cure rate between CHM formula granules and placebo based on conventional therapy.

Study ID	Treatment		Placebo		P value
	n/N	%	n/N	%	
*Chronic Hepatitis B: HBV DNA negative after 48-week treatment*					
Zhan BL 2013	24/30	80.00	16/30	53.33	0.07

*Chronic Hepatitis B: HBeAg negative after 48-week treatment*					
Ye YA 2012	83/280	29.64	50/280	17.86	0.001
Zhan BL 2013	14/30	46.67	6/30	20.00	0.04
Meta-analysis RR and 95% CI: 1.73 [1.30,2.31]; I^2^ = 0%					

*Chronic Hepatitis B with YMDD: HBV DNA negative after 48-week treatment*					
Xie WN 2016	7/36	19.44	1/33	3.03	0.07

*Chronic Hepatitis B with YMDD: HBeAg negative after 48-week treatment*					
Xie WN 2016	9/36	25.00	0/31	0.00	0.05

*Herpes simplex keratitis: Symptoms disappeared and normal laboratory test*					
Song JK 2016	24/33	72.73	14/31	45.16	0.03
Song JK 2016a	23/34	67.65	13/32	40.63	0.04

*Depression: The score of HAMD-17 <7*					
Liu J 2012	12/32	37.50	8/33	24.24	0.25

*Myasthenia gravis: Reduction of the score of myasthenia gravis scale≥95*%					
Shuang XP 2014	0/20	0.00	0/18	0.00	-

**Table 4:** Comparison of the effectiveness rate between CHM formula granules and placebo.

Study ID	Treatment		Placebo		P value
	n/N	%	n/N	%	
*HBV carrier: Reduction of within-group HBV DNA levels by more than 2lgIU/ml*					
He JS 2012	38/174	21.84	5/93	5.38	0.001
Li HZ 2012	37/191	19.37	4/94	4.26	0.002
Ni W 2015	6/40	15.00	2/20	10.00	0.59
Peng DT 2016	15/44	34.09	2/23	8.70	0.04
Xing YF 2012	37/191	19.37	9/187	4.81	<0.001
Meta-analysis RR and 95% CI: 4.64 [2.89,7.45]; I^2^ = 0%					

*Non-alcoholic fatty liver disease: Reduction in ALT by >50% after 12-week treatment*					
Cheng Y 2017	33/40	82.50	21/40	52.00	0.006

*Depression: Reduction in Edinburgh Postnatal Depression Scale (EPDS)≥50*%					
Xu F 2013-1	40/50	80.00	24/50	48.00	0.001
Xu F 2013	35/45	77.78	18/45	40.33	0.0004

*Fever: Reduction in fever, and resolution of some cold symptoms*					
Cheng Y 2016	39/41	95.12	26/40	65.85	0.003
Xie JH 2017	45/48	93.75	44/48	91.67	0.7
Meta-analysis(random) RR and 95% CI: 3.78 [0.50,28.36]; I^2^ = 70%					

*Migraine: Reduction in frequency/duration of headaches by >50% after 12-week treatment*					
Fu CH 2014	101/129	78.29	49/64	76.56	0.79
Fu CH 2014	92/129	71.32	46/64	71.88	0.94

*AIDS: Increase in CD4 levels by >30*%* after 6-month treatment*					
Li Y 2012	14/36	38.89	6/36	16.67	0.04

**Table 5:** Comparison of the effectiveness rate between CHM formula granules and placebo based on conventional therapy.

Study ID	Treatment		Placebo		P value
	n/N	%	n/N	%	
*COPD: the comprehensive improvement of pulmonary function, dyspnoea classification, 6-min walking distance and BMI*					
Chen SQ 2017(stable period)	56/60	93.33	48/60	80.00	0.04
Xie WY 2017(acute period)	57/60	95.00	53/60	88.33	0.19
Shang LZ 2017	92/100	92.00	80/100	80.00	0.02
Meta-analysis RR and 95% CI: 1.13 [1.06,1.22]; I^2^ = 0%					

*Diabetic peripheral neuropathy: Pain relief and neurological function improvement*					
Hu WH 2015	42/48	87.50	33/47	70.21	0.04

*Depression: Reduction in HAMD Scale ≥25*%					
Liu J 2012	28/32	87.50	25/33	75.76	0.23

*Angina pectoris: Pain relieved, attacks reduced, ECG improved, nitro-glycerine reduced by 50*%*-80*%					
Pang LJ 2013	22/29	75.86	16/24	66.67	0.47

*Myasthenia gravis: Reduction of the score of myasthenia gravis scale≥25*%					
Shuang XP 2014	18/20	90.00	16/18	88.89	0.91

*Herpes simplex keratitis: Symptoms improved and almost normal laboratory test*					
Song JK 2016	33/33	100.00	31/31	100.00	1.00
Song JK 2016a	34/34	100.00	30/32	93.75	0.23

**Table 6:** The overview of the effectiveness of CHM formula granules only.

Diseases	No. of studies	Cure rate		Effectiveness rate	
		CHM granules	Placebo	CHM granules	Placebo
HBV carrier/recrudescence HBV DNA (HBeAg)	5	3.1% (7.4%)	0.5% (3.8%)	20.8%	5.3%*∗*
Chronic hepatitis C HCV RNA	3	16.9%	3.0%*∗*	-	-
Non-alcoholic fatty liver	1	47.5%	25.0%*∗*	82.5%	52.5%*∗*
Depression	2	28.4%	10.5%*∗*	78.9%	44.2%*∗*
Fever	2	62.9%	40.0%*∗*	94.4%	87.5%
Migraine	1	4.2%	0.0%	74.8%	74.2%
AIDS	1	-	-	38.9%	16.7%*∗*

*∗*P<0.05 (result of individual study or meta-analysis).

**Table 7:** The overview of the effectiveness of CHM formula granules based on conventional therapy.

Diseases	No. of studies	Cure rate		Effectiveness rate	
		CHM granules	Placebo	CHM granules	Placebo
Chronic Hepatitis B HBV DNA (HBeAg)	3	47.0% (30.6%)	28.0% (16.2%*∗*)	-	-
Myasthenia gravis	1	0.0%	0.0%	90.0%	88.9%
Herpes simplex keratitis	1	70.1%	42.9%*∗*	100.0%	96.8%
Depression	1	37.5%	24.2%	87.5%	75.8%
COPD	3	-	-	93.2%	82.3%*∗*
Diabetic peripheral neuropathy	1	-	-	87.5%	70.2%*∗*
Angina pectoris	1	-	-	75.9%	66.7%

*∗*P<0.05 (result of individual study or meta-analysis).
